# Cases of Sudden Death Attributed to Imprudent Bathing

**Published:** 1859-05

**Authors:** Edward Warren

**Affiliations:** Newton Lower Falls


From the Boston Medical and Surgical Journal.

*Case of Sudden Death, Attributed to Imprudent Bathing.* By Edward Warren, M.D.

A Singular case of sudden death came under my notice upon Thursday last, the 17th inst.

I was called, about halfpast four o'clock in the afternoon, to visit a boy in a fit. On arriving at the house, I found a lad of 9 years of age in severe convulsions of a peculiar character. I learnt from the young man who was with him, and who drove a milk wagon, that he belonged to Newtonvile, and had been in the frequent habit of getting into his cart upon the road, for the pleasure of a ride. On this day, on passing the usual place, he whistled and checked the speed of his horse. to give him the opportunity, if he wished. The boy came out of the woods, ran after the wagon, and got in. He talked for some time, and told the milkman that he had been into water, which the latter supposed was said in joke. He continued to talk until they reached Weston bridge, a few rods from a house were they stopped to take milk. When his companion first noticed that anything was amiss, the appearance of the lad led him to suppose he was seized with lockjaw. He was carried immediately into the house, vomiting by the way. I saw him very soon after. There was, at first some relaxation of the spasms; and although his teeth were firmly closed together, I found no great difficulty in getting down a dose of ipecac. Sinapisms were applied to the bowels, and the limbs and head rubbed with ammonia. His feet had already been placed in a warm bath.

The spasms became more violent; they were of a tetanic character. The facial muscles were in powerful action, producing every variety of grimace and contortion of the countenance, resembling the action of galvanism upon the recent subject. The pupils of the eyes weie fixed and turned downwards, so as to be scarcely visible. There was frothing at the mouth. The body was bent backward, producing opisthotonos. The whole appearance resembled that which I have witnessed from an overdose of strychnine, and led me to inquire whether it was possible he had found any poisonous berries in the woods.

As soon as sufficient hot water could be obtained, I had him placed in a hot bath. The limbs became relaxed, the pupils assumed a natual position and there was slight appearance of returning consciousness. But the contortions of the facial muscles soon returned. Gradually, however, they ceased, the whole system became relaxed, and the pulse impercedtible; the breathing became more gentle, then intermitted, and soon stopped. His death took place within about an hour and a half from the commencement of the attack. The sinapisms had produced no redness, and there was no effect from the ipecac.

I subsequently learnt that he, with too other boys, had gone into a pond to bathe. One of the others had gone home directly, and dropped down insensible upon reaching his father's yard. He recovered. The third boy, it is said, did not take off his clothes as the others did, but merely waded into the water. I am told also, that they had dug up and eaten some roots which they found in the woods, and that one of these roots was shown to a physician in Newtonville, who pronounced it of a medicinal character, but not poisonous.

I am not aware of any root or berry in the neighborhood, that could produce effects resembling those from nux vomica. On the other hand, it is singular for chill to act in this manner upon the spinal marrow and brain, producing the effect of an irritant and stimulant. The usual action of cold is to torpify, not excite, the nervous system. Torpor and insensibility are the well known results of cold. Paralysis of the lower limbs is not an unfrequent attendant upon immersion, for some time, in cold river or brook water. We sometimes hear of ice being broken in winter, for the purpose of religious immersion. No unfavorable results have occurred in these cases. But the vomiting, and the high degree of the nervous centres, is at least uncommon.

The patient ran after the milk wagon, got in as usual, and appeared lively and talkative. It might be supposed that the run after the wagon would counteract, in some degree, the effect of cold bathing, by exciting the circulation; while the subsequent ride was not long enough to produce much chill; the weather on the 18th not being very cold. If the symptoms could have been attributed to any fresh poisonous root, there was just time enough for its influence upon the system, which the exercise of running and riding might have accelerated.

The *Traveller* states that he was buried upon Sunday, and that his body presented a remarkably fresh appearance, the color hovering about the lips, as if life still lingered. Such I believe to be frequenly the case where life is destroyed by sudden and powerful action upon the nervous system.

*Newton Lower Falls, March* 24, 1859.

